# Zinc Regulates Meiotic Resumption in Porcine Oocytes via a Protein Kinase C-Related Pathway

**DOI:** 10.1371/journal.pone.0102097

**Published:** 2014-07-14

**Authors:** Ming-Hui Zhao, Jung-Woo Kwon, Shuang Liang, Seon-Hyang Kim, Ying-Hua Li, Jeong-Su Oh, Nam-Hyung Kim, Xiang-Shun Cui

**Affiliations:** 1 Department of Animal Sciences, Chungbuk National University, Cheongju, Republic of Korea; 2 Brain Korea 21 Center for Bio-Resource Development, Cheongju, Republic of Korea; 3 Department of Genetic Engineering, College of Biotechnology and Bioengineering, Sungkyunkwan University, Suwon, Republic of Korea; Institute of Zoology, Chinese Academy of Sciences, China

## Abstract

Zinc is an extremely important trace element that plays important roles in several biological processes. However, the function of zinc in meiotic division of porcine oocytes is unknown. In this study, we investigated the role of zinc during meiotic resumption in *in vitro* matured porcine oocytes. During meiotic division, a massive release of zinc was observed. The level of free zinc in the cytoplasm significantly increased during maturation. Depletion of zinc using N, N, N′, N′-tetrakis (2-pyridylmethyl) ethylenediamine (TPEN), a Zn^2+^ chelator, blocked meiotic resumption in a dose dependent manner. The level of phosphorylated mitogen activated protein kinase (MAPK) and p34^cdc2^ kinase activity were reduced when zinc was depleted. Moreover, zinc depletion reduced the levels of phosphorylated protein kinase C (PKC) substrates in a dose dependent manner. Real-time PCR analysis showed that expression of the MAPK- and maturation promoting factor related genes *C-mos*, *CyclinB1*, and *Cdc2* was downregulated following zinc depletion. Treatment with the PKC agonist phorbol 12-myristate 13-acetate (PMA) increased phosphorylation of PKC substrates and MAPK and increased p34^cdc2^ kinase activity. This rescued the meiotic arrest, even in the presence of TPEN. Activation of PKC by PMA increased the level of zinc in the cytoplasm. These data demonstrate that zinc is required for meiotic resumption in porcine oocytes, and this appears to be regulated via a PKC related pathway.

## Introduction

During mammal oogenesis, follicle enclosed oocytes arrest at prophase I during the first meiosis. Meiosis resumes once oocytes are released from follicles or are stimulated by specific stimuli, such as gonadotropins. During and following resumption of the first meiosis, chromatin starts to condense, proteins within germinal vesicles (GV) are phosphorylated, and GV breakdown (GVBD).

Meiotic resumption is regulated by several protein kinases. In porcine oocytes, meiotic resumption is facilitated by maturation promoting factor (MPF), a heterodimeric protein composed as Cyclin B1 and Cdc2 (also known as cyclin dependent kinase (CDK)-1 or p34^cdc2^) [1]. Microinjection of heterologous maturating cytoplasm into immature porcine oocytes results in GVBD after 8 h of culture [2], whereas inhibition of MPF activation blocks GVBD. Although Cyclin B1 and Cdc2 are associated with each other in GV stage oocytes [3], the increase in the level of active MPF requires protein synthesis [4].

Mitogen activated protein kinase (MAPK) is also important for meiosis. MAPK is inactive in GV stage porcine oocytes, and is activated around the time of GVBD [5], [6]. Activation of MAPK is regulated by the upstream kinase c-mos. The MAPK inhibitors PD98059 and U0126 significantly reduce MAPK activity and prevent GVBD in cumulus enclosed oocytes [7], [8].

Protein kinase C (PKC), which is zinc finger content kinase, play important role in modulating meiotic resumption. Activation of PKC inhibits GVBD in denuded mouse oocytes [9], but enhances the spontaneous maturation rate of porcine oocytes [10], and the meiotic resumption in porcine oocyte which is associated with the PKC pathway activation in cumulus cells [11].

Regulation of cell cycle by PKC closely related with MPF and MAPK. The two subunit of MPF, Cyclin B1 and Cdc2, were shown to be included in the substrates of PKC [12]. Treatment of late MI stage oocytes with the PKC inhibitor, bisindolylmaleimide I (BIM), transiently reduced M phase promoting factor (MPF) activity [13]. MAPK also be regulated by PKC. The regulation of MAPK activity by PKC might be involved in the regulation of cAMP mediated MAPK activation [14].

Several recent reports have demonstrated unequivocally that elemental zinc is necessary for meiotic progression [15]–[18]. Total zinc, including zinc bound to proteins, is extremely abundant in oocytes prior to maturation [19]. Zinc is involved in preventing premature GVBD in GV stage mouse oocytes and the level of zinc was increased during maturation of mouse oocytes [19]. Zinc is also required for the stability of the EMI2/APC complex to prevent MPF being ubiquitinised [18]. Although the acceleration of GVBD in mouse oocytes following zinc depletion is dependent on the Mos-MAPK pathway [17], the specific regulation mechanism by zinc in this pathway in mammalian oocytes is still unclear.

Several studies have investigated the function of zinc in meiotic division of mouse oocytes [15], [17], [20]; however, little information is available on porcine oocyte. In this study, we investigated the role of zinc during meiotic resumption in *in vitro* matured porcine oocytes by depleting zinc. Our results revealed that zinc is essential for meiotic resumption, and, more importantly, that zinc regulates meiotic resumption via modulating the activity of PKC, which, in turn, affects the activities of MAPK and MPF. Moreover, this study shows that PKC is important for the regulation of zinc content in oocytes.

## Materials and Methods

Unless otherwise indicated, all chemicals were purchased from Sigma Chemical Company (Sigma–Aldrich, St. Louis, MO, USA).

### Collection of porcine oocytes and in vitro maturation (IVM)

Ovaries from pre-pubertal gilts were collected from a local slaughterhouse. Cumulus oocyte complexes (COCs) were aspirated from follicles that were 3–6 mm in diameter. COCs that were surrounded by a minimum of three layers of cumulus cells were selected for analysis. COCs were washed three times in TL-HEPES supplemented with 10% polyvinyl alcohol (PVA) and 0.05 g/L gentamycin. COCs were washed three times in IVM medium (TCM-199 supplemented with 0.1 g/L sodium pyruvate, 0.6 mM L-cysteine, 10 ng/mL epidermal growth factor, 10% porcine follicular fluid, 10 IU/mL luteinizing hormone, and 10 IU/mL follicle stimulating hormone) and were then transferred to IVM medium. To observe the effects of TPEN and the PKC pathway on the maturation of porcine oocytes, various concentrations of TPEN, PMA, and staurosporine were added to the medium. At the end of the experiment, cumulus cells were removed by pipetting with 0.1% hyaluronidase. Denuded oocytes were collected for nuclear staining or another experiment.

### Nuclear staining of porcine oocytes

To observe the effects of TPEN, PMA, and staurosporine on meiotic resumption in porcine oocytes, denuded oocytes were fixed in 3.7% (v/v) formaldehyde at room temperature for 20 min after 25 h of IVM, and were then stained with 10 µg/mL Hoechst 33342 for another 5 min. After washing three times in PBS containing 0.1% (w/v) PVA, oocytes were mounted and observed under ultraviolet light using a fluorescence microscope (Nikon, Japan). The meiotic stage of the oocytes was assessed as described previously [21], [22]. Briefly, oocytes with diakinesis nuclei were judged to be at GVBD stage, oocytes with chromosomes arranged at the equator were judged to be in metaphase (M) I, and oocytes with polar bodies were judged to be in MII.

### Measurement of zinc using the fluozin-3-AM indicator dye

Relative zinc content was measured using fluozin-3-AM according to a previously described method [23]. Briefly, COCs cultured in IVM medium were denuded and loaded into 20 µl of medium containing fluozin-3-AM (2 µM, Invitrogen, excitation/emission: 494 nm/516 nm) for 2 hours at 37°C. Oocytes were then washed briefly in fresh medium, mounted onto glass slides with etched rings to prevent rupture of the oocytes, and covered with a coverslip. Immediate imaging provided the most reproducible results, although the signal was extremely stable with little bleaching for up to 30 min of imaging. The pixel intensity per unit area after background subtraction was determined following culture for different amounts of time using a Nikon fluorescence microscope (Nikon, Japan). Fluozin-3-AM has been extensively characterized to measure free intracellular zinc in living cells using microscopy, and has an affinity constant for zinc of 15 nM.

### Real-time PCR with SYBR green

Following IVM, mRNA was isolated from porcine oocytes using the Dynabeads mRNA Direct Kit (Dynal Asa, Oslo, Norway) according to the manufacturer's instruction. First-strand cDNA was synthesized by reverse transcribing mRNA using the Oligo (dT)_12–18_ primer and SuperScript TM III Reverse Transcriptase (Invitrogen Co., Grand Island, NY). Real-time PCR was performed using a CFX96 Touch Real-time PCR Detection System (Bio-Rad) in a final reaction volume of 20 µl, including SYBR Green, a fluorophore that binds to double stranded DNA (qPCR kit from FINNZYMES, Finland). The PCR conditions were as follows: 95°C for 10 min followed by 39 cycles of 95°C for 30 sec, 60°C for 30 sec, and 72°C for 25 sec, and a final extension at 72°C for 5 min. Finally, gene expression was quantified using the 2-ddCt method, and mRNA levels were normalized against that of *Gapdh*. The primers used to amplify each gene are shown in Table 1.

**Table 1:** Primer used for real-time PCR.

Gene	Gene Bank accession number	Primer sequence (5′-3′)	Length (bp)
*Cdc2*	AB 045783	F: GGGCACTCCCAATAATGAAGT	260
		R: GTTCTTGATACAACGTGTGGGAA	
*Cycling B1*	L48205	F: GCTCCAGTGCTCTGCTTCTC	177
		R: ACAAACTTTATTAAAAGTAAATAAGTG	
*C-mos*	NM _001113219	F: TGGGAAGAAACTGGAGGACA	121
		R: TTCGGGTCAGCCCAGTTCA	
*Gapdh*	AF 017079	F: GGGCATGAACCATGAGAAGT	230
		R: AAGCAGGGATGATGTTCTGG	

### Western blotting

For the PKC substrate assay, 200–400 oocytes were transferred to Laemmli sample buffer and incubated at 95°C for 5 min. The total protein content was subsequently separated by electrophoresis through a Mini-PROTEAN TGXTM Precast Gel for 2 h at 100 V. Proteins were then transferred to a polyvinylidene fluoride membrane. After blocking with Tris buffered saline containing 5% bovine serum albumin for 1 h, the membrane was incubated with a primary antibody against phospho-(Ser) PKC substrates (Cell Signaling Technology, Tokyo, Japan) for 1 h at room temperature, and then with horseradish peroxidase conjugated anti-rabbit IgG for 1 h. Finally, bands were visualized using Enhanced Chemiluminescence Luminol Reagent (ECL).

### p34^cdc2^ kinase activity assay

p34^cdc2^ kinase activity was measured using a Mesacup cdc2 Kinase Assay Kit (code no. 5235; MBL, Nagoya, Japan) according to a previously described method [24]. Using this method, the correlation coefficient between p34^cdc2^ kinase activity (as determined using the Mesacup cdc2 Kinase Assay Kit) and histone H1 kinase activity (as measured by detection of radioactivity) can be as high as 0.9961.

Briefly, 5 µl of oocyte extract (containing 20 oocytes) was mixed with 45 µl of kinase assay buffer B, which comprised 25 mM HEPES buffer (pH 7.5; MBL), 10 mM MgCl_2_ (MBL), 10% (v/v) MV peptide solution (SLYSSPGGAYC; MBL), and 0.1 mM ATP (Sigma). The mixture was incubated for 30 min at 30°C. The reaction was terminated using 200 µl PBS containing 50 mM EGTA (MBL). Phosphorylation of MV peptides (Dynal Biotech Asa, Oslo, Norway) was detected using an enzyme-linked immunosorbent assay (ELISA). Values were expressed as optical densities (ODs). Each independent experiment was repeated three times.

### Statistical analysis

All data were analyzed using SPSS software (version 11.0, USA). Flouzin-3 signal intensity, phospho-PKC substrates content, gene expression and p34^cdc2^ kinase activity were analyzed by one-way ANOVA. Percentages of oocytes that developed to a particular stage were determined by Chi-square procedures. P<0.05 was considered significant.

### Experimental design

#### Experiment 1

Zinc content during oocyte maturation. To examine the level of zinc during oocyte maturation, porcine oocytes were stained with fluozin-3-AM after 0, 20, 28, and 44 h of IVM, when oocytes are at the GV, GVBD, MI, and MII stages [25], [26], respectively. The fluorescence intensity of fluozin-3-AM, indicative of the zinc content, at 0 h was set to 1. The relative fluorescence intensities of fluozin-3-AM were then compared among the oocytes at the various stages.

#### Experiment 2

Effect of zinc depletion on meiotic resumption in porcine oocytes. Zinc was depleted from porcine oocytes by treatment with 0.1, 1.0, 2.0, 2.5, or 3.0 µM TPEN, or alternatively oocytes were not treated. Nuclear morphology was examined after 25 h of IVM, when almost all oocytes in the untreated group were at the GVBD stage. The percentages of TPEN treated oocytes at the GV, GVBD, MI, and MII stages were determined.

#### Experiment 3

Effect of TPEN concentration on polar body extrusion in porcine oocytes. To determine the concentration of TPEN to be used in Experiment 4, porcine oocytes were cultured in IVM medium containing 0, 2.5, 2.7, 3.0, or 4.0 µM TPEN. The percentage of oocytes that had extruded a polar body was examined after 44 h of IVM.

#### Experiment 4

Effect of TPEN on polar body extrusion in porcine oocytes. In Experiment 3, treatment with 2.7 µM TPEN reduced the maturation rate to about 50%. To confirm that TPEN modulates meiotic resumption in porcine oocytes, oocytes were treated with or without 2.7 µM TPEN for the first 20 h of IVM. Oocytes were subsequently cultured in IVM medium lacking TPEN. The percentage of oocytes that had extruded a polar body was examined after 28, 31, 34, 37, and 40 h of IVM.

#### Experiment 5

Effect of zinc depletion on expression of MPF- and MAPK- related genes. To investigate why TPEN treatment affects meiotic arrest, the mRNA levels of the MPF- and MAPK related genes *Cdc2*, *CyclinB1*, and *C-mos* in oocytes treated with or without 2.7 µM TPEN were determined by real-time PCR after 25 h of IVM.

#### Experiment 6

Effect of a PKC inhibitor and activator on meiotic resumption and the zinc content in oocytes. To investigate whether zinc regulates meiotic resumption via the PKC pathway, oocytes were cultured in the presence of various concentrations of staurosporine (0, 1, 10, 100, or 1000 nM) and meiotic resumption was examined after 25 h of IVM.

Oocytes were cultured in the presence of 2.7 µM TPEN and/or 100 nM PMA. Nuclei of oocytes were examined after 25 h of IVM. To confirm the relationship between zinc and PKC activity, oocytes were treated with 100 nM PMA for 1 h prior to IVM, and free zinc in oocytes was monitored using fluozin-3-AM.

#### Experiment 7

Effect of zinc depletion and PMA treatment on phosphorylation of PKC substrates and MAPK. To confirm the relationship between zinc and PKC, phosphorylation of PKC substrates and MAPK in oocytes treated with 2.7 µM TPEN and/or 100 nM PMA for 25 h of IVM were examined by Western blotting. Phosphorylation of PKC substrates was also examined by Western blotting in oocytes treated with various concentrations of TPEN (0, 1.0, 2.0, or 3.0 µM) or with staurosporine for 25 h of IVM.

#### Experiment 8

Effect of zinc depletion and PMA treatment on p34^cdc2^ kinase activity. MPF activity was assessed by examining p34^cdc2^ kinase activity using an ELISA after 25 h of IVM in oocytes treated with 2.7 µM TPEN and/or 100 nM PMA.

## Results

### Level of zinc in porcine oocytes during IVM

**Figure 1:**
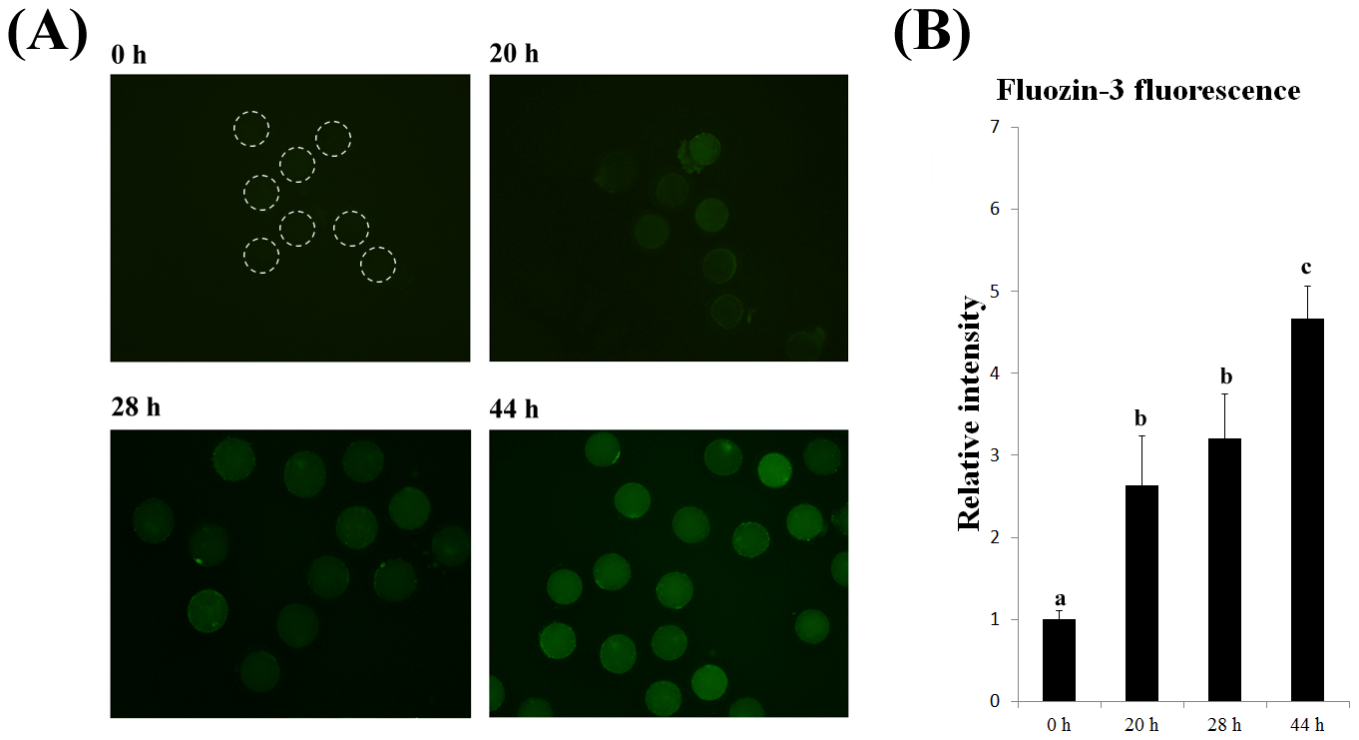
The level of free cytoplasmic zinc in oocytes increases during maturation. (A) Representative images of oocytes after 0, 20, 28, and 44 h of IVM. (**B**) The fluorescence intensity of fluozin-3-AM relative to that in oocytes after 0 h of IVM (set at 1) was analyzed using Photoshop. Data are expressed as mean values ± standard error of the mean (SEM) of three independent experiments. White lines indicate the peripheries of oocytes. Different letters above the bars denote statistically significant differences (P<0.05).

To examine the levels of zinc in porcine oocytes during maturation, we determined the fluorescence intensity of fluozin-3-AM in denuded oocytes after 20, 28, and 44 h of IVM relative to that in denuded oocytes at 0 h. The relative fluorescence intensity of fluozin-3-AM was significantly higher (P<0.05) in GVBD stage oocytes than in GV stage oocytes. And significantly higher (P<0.05) in MII stage oocytes than in MI stage oocytes, but did not significantly differ between GVBD stage and MI stage oocytes. (Figure 1 A and B).

### Effect of zinc depletion on meiotic resumption in porcine oocytes

**Figure 2:**
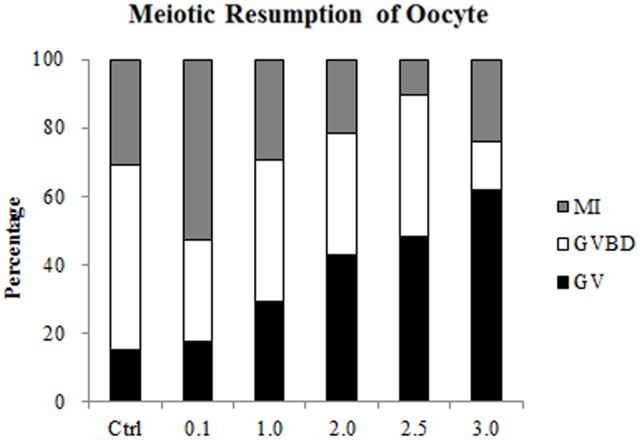
Effects of different concentrations of TPEN on meiotic resumption in porcine oocytes. Nuclear distribution was assessed after 25(0, 0.1, 1.0, 2.0, 2.5, and 3.0 µM). The percentages of oocytes at the GV, GVBD, and MI stages are shown. TPEN inhibits oocyte maturation in a dose-dependent manner. Data are presented as mean values of three independent experiments. Ctrl, control group.

To investigate the effect of zinc depletion on meiotic resumption in porcine oocytes arrested at prophase I, free zinc in these oocytes was depleted by treatment with TPEN. After 25 h of IVM, TPEN treatment induced the arrest of porcine oocytes at GV stage in a dose dependent manner. The percentages of oocytes arrested at GV stage were significantly higher in groups treated with 1.0 µM (30.68±2.95%, n = 113), 2.0 µM (42.86±4.59%, n = 95), 2.5 µM (48.42±3.03%, n = 84), and 3.0 µM (60.85±5.15%, n = 92) TPEN than in the control group (15.60±0.20%, n = 83, P<0.05). (Figure 2).

### Effects of various concentrations of TPEN on polar body extrusion in porcine oocytes

**Figure 3:**
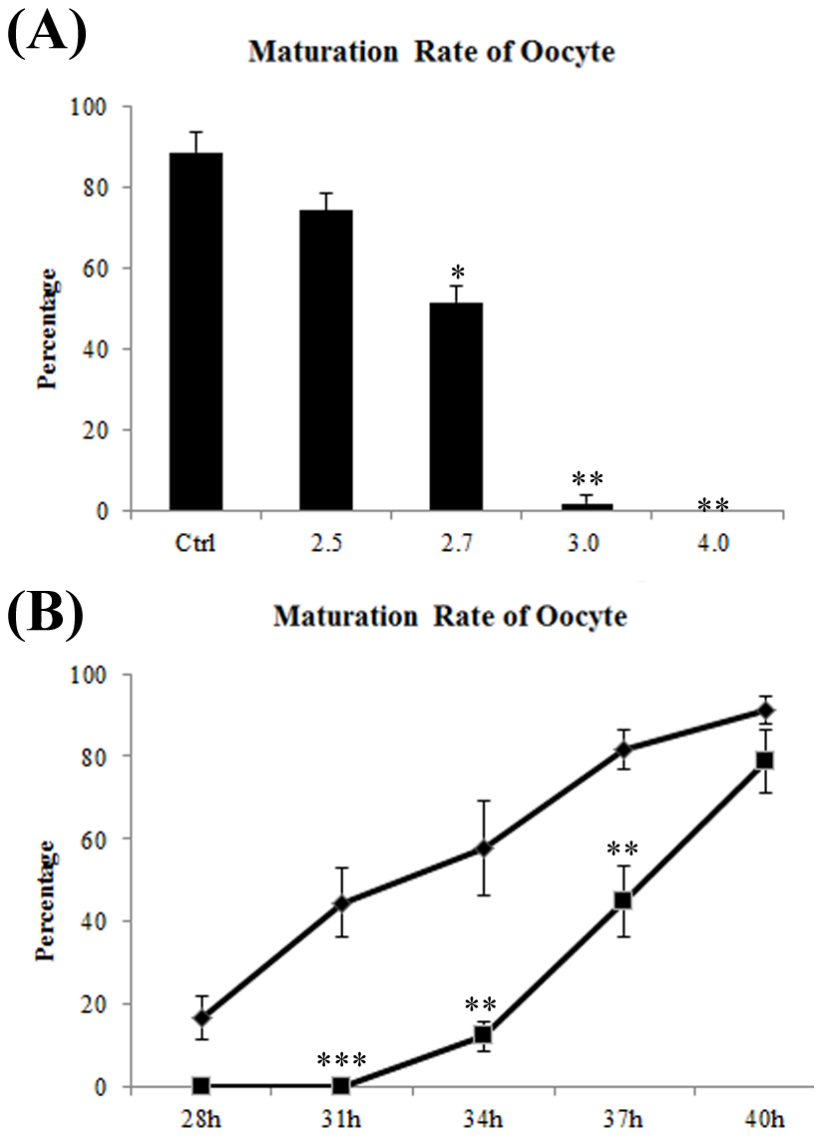
Effects of different concentrations of TPEN on the maturation of porcine oocytes. (A) TPEN inhibits oocyte maturation in a dose-dependent manner. Oocytes were cultured in IVM media containing various concentrations of TPEN, and the maturation rate was determined. The EC_50_ of TPEN in oocytes is about 2.7 µM. (**B**) Oocytes underwent IVM in the presence of TPEN for 20 h and were then transferred to media lacking TPEN. The maturation rate was determined. Data are presented as mean values ± SEM of three independent experiments. ⧫ Ctrl, control group, ▪ TPEN rescue group. *, P<0.05; **, P<0.01.

To determine the half maximal effective concentration (EC_50_) of TPEN in porcine oocytes, oocytes were cultured in IVM media containing various concentrations of TPEN (Figure 3A). The maturation rate did not significantly differ between oocytes cultured in in the absence of TPEN and those cultured in the presence of 2.5 µM TPEN (74.27±4.17%, n = 223). However, the maturation rate decreased sharply when oocytes were cultured with higher concentrations of TPEN. The maturation rate was significantly lower when oocytes were cultured with 2.7 µM (51.64±3.90%, n = 187) or 3 µM (1.83±1.13% n = 228, P<0.05) TPEN than when they were cultured in the absence of TPEN (88.44±4.90%, n = 166, P<0.05) (Figure 3A). Therefore, oocytes were treated with 2.7 µM TPEN in subsequent experiments.

Porcine oocytes underwent IVM in the presence of TPEN for 20 h and were then transferred to IVM media lacking TPEN, the so-called ‘TPEN rescue’ group. Although TPEN inhibited meiotic resumption, oocytes could extrude the first polar body (Figure 3B). After 31 h of IVM, the percentage of oocytes that had extruded a polar body was significantly higher in the control group than in the TPEN rescue group (57.59±6.59%, n = 75 vs. 0.00%, n = 74; P<0.01). The maturation rate of the TPEN rescue group after 34 h (12.06±4.17%, n = 70 vs. 57.59±7.23%, n = 76; P<0.05) and 37 h (44.76±5.45%, n = 72 vs. 81.64±5.98%, n = 75; P<0.05) of IVM was significantly lower than that of the control group. However, there were no significant difference after 40 h of IVM (control group 91.32±4.31%,n = 70 vs. TPEN rescue group 78.80±7.55%, n = 71; P>0.05). These results show that TPEN treatment significantly delayed the maturation of porcine oocytes.

### Effect of zinc depletion on the expression of maternal genes in porcine oocytes

**Figure 4:**
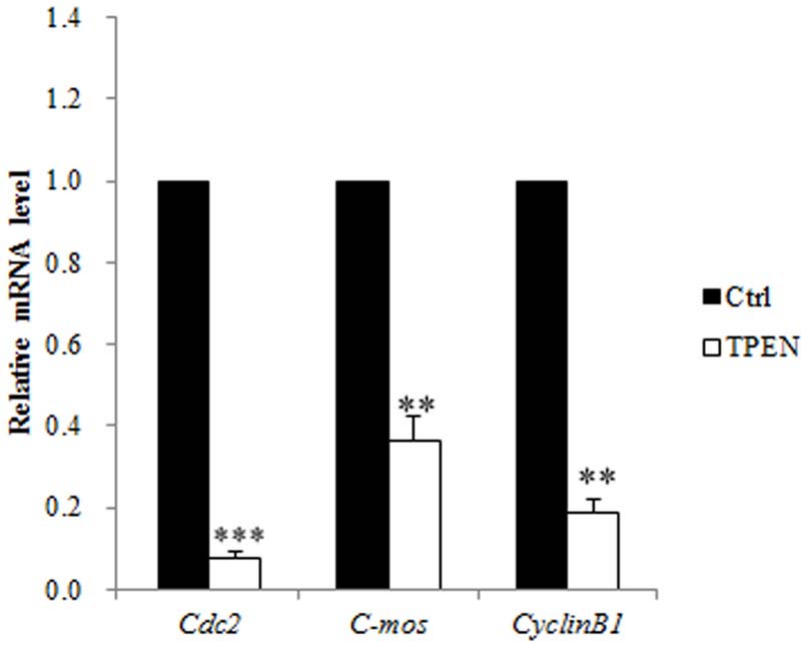
TPEN treatment affects the expression of maternal genes in porcine oocytes. TPEN treatment significantly decreases the mRNA levels of the MPF pathway-related genes *Cdc2* and *CyclinB1* and the MAPK pathway-related gene *C-mos*. Data are expressed as mean values ± SEM of three independent experiments. Ctrl, control group. **, P<0.01; ***, P<0.001.

Maternally inherited cytoplasmic genes are important for oocyte maturation and early embryo development. To investigate the molecular mechanism underlying how zinc depletion affects oocyte maturation, expression of the maternal genes *C-mos*, *Cyclin B1* and *Cdc2*, was examined in porcine oocytes. After 44 h of IVM, expression of *C-mos* (P<0.01), *CyclinB1* (P<0.01), and *Cdc2* (P<0.001) was significantly lower in TPEN treated oocytes than in control oocytes (Figure 4).

### Effect of a PKC inhibitor and activator on meiotic resumption in porcine oocytes

**Figure 5:**
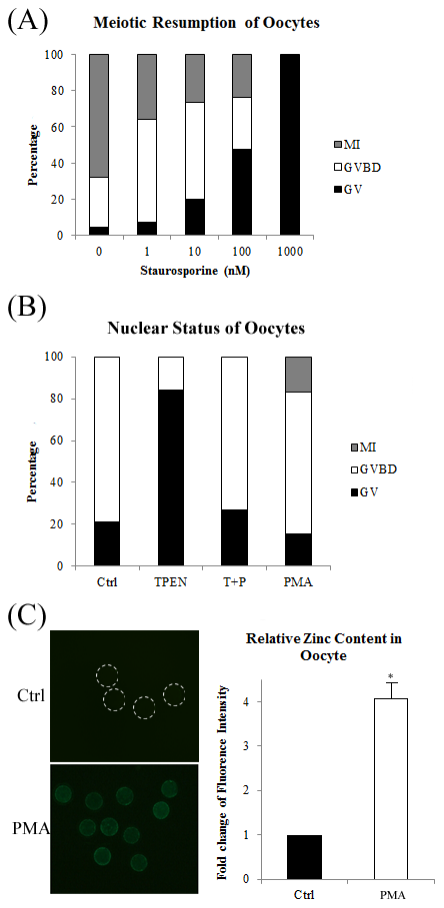
TPEN controls meiotic resumption in a PKC-dependent manner. (A) Nuclear distribution was examined after 25 h of IVM and treatment with various concentrations of staurosporine. The percentages of oocytes at the GV, GVBD, and MI stages are shown. (**B**) Nuclear distribution was examined after 25 h of IVM and treatment with TPEN and/or PMA. The percentages of oocytes at the GV, GVBD, and MI stages are shown. (**C**) Change in the level of free zinc following PMA treatment. Zinc content was analyzed using Photoshop and was normalized against the level in the control group (set at 1). Ctrl, control group; TPEN, oocytes matured in the presence of 2.7 µM TPEN; T+P, oocytes matured in the presence of TPEN and PMA; PMA, oocytes matured in the presence of PMA only. PMA (in figure C), GV-stage oocytes treated with PMA for 1 h. White dashed lines indicate the peripheries of oocytes. Data are expressed as mean values ± SEM of three independent experiments. *, P<0.05.

To clarify the mechanism underlying how zinc regulates meiotic resumption, we examined the relationship between zinc and PKC. The PKC inhibitor staurosporine inhibited meiotic resumption in porcine oocytes in a dose dependent manner, similar to TPEN. Treatment with 100 nM staurosporine significantly increased the percentage of oocytes arrested at GV stage (20.80±3.87%, n = 58 vs. 2.96±2.57%, n = 69 in control oocytes; P<0.05). Treatment with 1000 nM staurosporine completely blocked meiotic resumption, with almost all oocytes arrested at GV stage (98.77±2.13%, n = 76 vs. 2.96±2.57%, n = 69 in control oocytes; P<0.01, Figure 5A).

We also examined whether PMA, a PKC activator, could rescue the meiotic arrest caused by TPEN. After 25 h of IVM, the percentage of oocytes at GV stage was significantly higher in the TPEN group than in the control group (83.89±11.94%, n = 62 vs. 21.54±6.80%, n = 60; P<0.01). However, this rate did not significantly differ between the TPEN plus PMA group (26.91±3.10%, n = 67) or the PMA group (15.39±6.94%, n = 79) and the control group (21.54±6.80%, n = 60, P>0.05, Figure 5B). Treatment with PMA for 1 h significantly increased (P<0.05) the cytoplasmic zinc content in GV stage oocytes by about 4 fold (Figure 5C).

### Effect of zinc depletion and PMA treatment on levels of phosphorylated PKC substrates and phosphorylated ERK1/2

**Figure 6:**
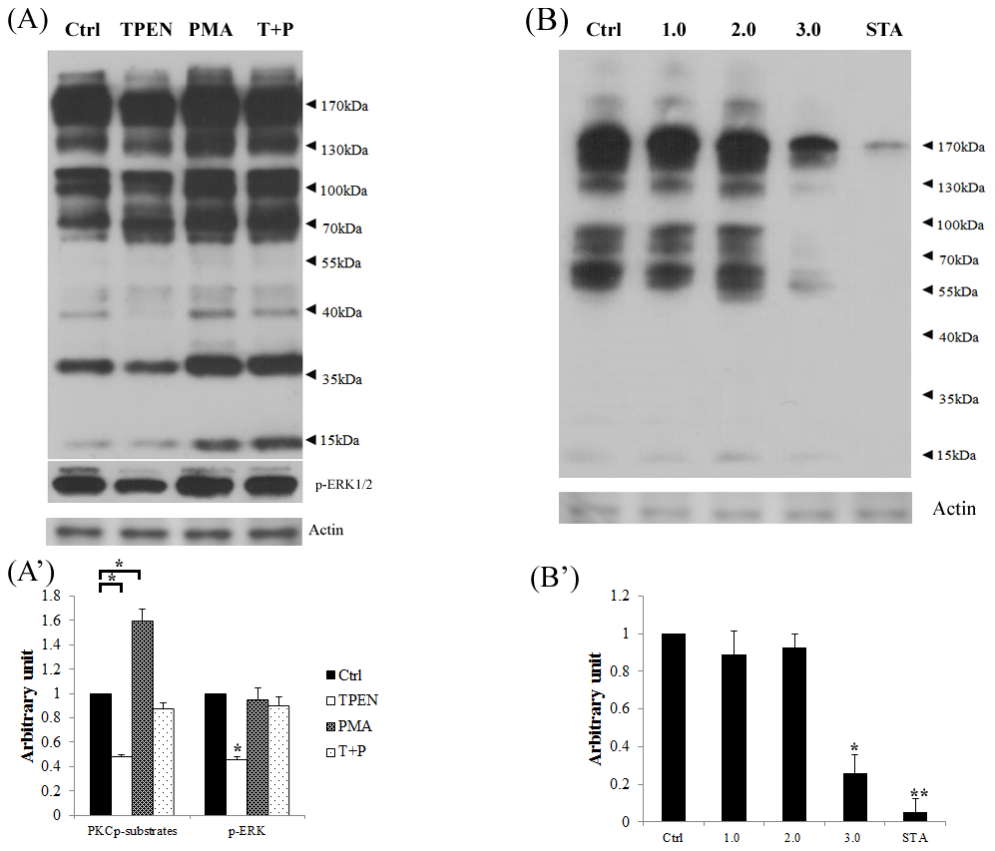
TPEN inhibits phosphorylation of PKC substrates in porcine oocytes. (A) TPEN downregulates phosphorylation of PKC substrates and MAPK in porcine oocytes, while PMA increases the phosphorylation of these proteins, even in the presence of TPEN. The density of band were digitalized. Oocytes were treated with TPEN, PMA, or TPEN plus PMA (T+P). A sample containing 400 oocytes was loaded per lane. (**B**) Oocytes were treated with various concentrations of TPEN (0, 0.1, 1.0, and 3.0 µM) or staurosporine (STA). Phosphorylation of PKC substrates was examined after 25 h of IVM. A sample containing 200 oocytes was loaded per lane. (A′) and (B′) Western blots were analyzed using Gel-pro software. The IOD of each treatment group was normalized against that of the control group. Ctrl, control group. Data are expressed as mean values ± SEM of three independent experiments. *, P<0.05; **, P<0.01.

To gain insight into the molecular mechanism underlying how zinc regulates meiotic resumption in oocytes, we directly assayed phosphorylated PKC substrates in porcine oocytes following zinc depletion. Results of western blotting analysis showed that zinc depletion significantly reduced (P<0.01) the phosphorylation of total PKC substrates, but the reduction was rescued by PMA (P<0.05, Figure 6A and A′). Phosphorylation of PKC substrates was significantly higher (P<0.05) in the PMA group than control and TPEN group. The pattern of phospho-ERK1/2 as similar as phospho-PKC substrates result. TPEN treatment significantly decreased the content of phospho-ERK1/2 (P<0.05), but the decrease was rescued by PMA. Treatment with low concentrations (1.0 or 2.0 µM) of TPEN did not inhibit phosphorylation of PKC substrates (Figure 6B and B′). However, treatment with 3.0 µM TPEN or staurosporine significantly reduced (P<0.05) phosphorylation of PKC substrates, in comparison to the control group.

### Effect of zinc depletion and PMA treatment on p34^cdc2^ kinase activity

**Figure 7:**
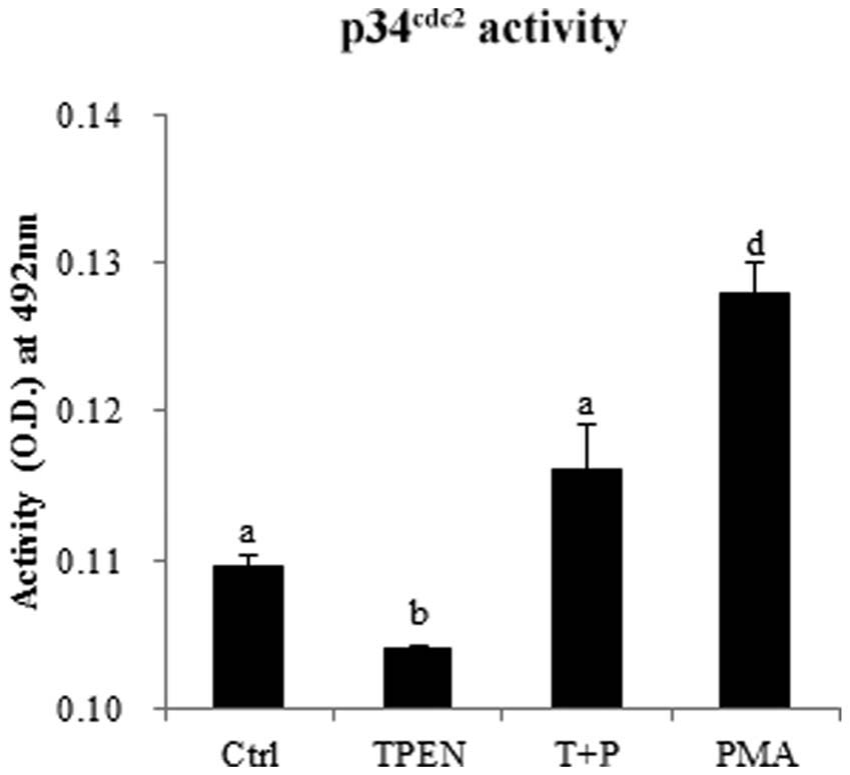
TPEN treatment reduces p34^cdc2^ kinase activity in porcine oocytes. p34^cdc2^ kinase activity was examined in oocytes after 25 h of IVM in the absence (Ctrl, control group) or presence of TPEN, PMA, or TPEN plus PMA (T+P). Twenty oocytes were examined per group. Data are expressed as mean values ± SEM. Different letters above the bars denote statistically significant differences (P<0.05).

The pattern of p34^cdc2^ kinase activity in oocytes was in agreement with levels of phosphorylated PKC substrates and the results were shown in Figure 7. Results indicated that the activity of p34^cdc2^ kinase in oocytes was significantly reduced by TPEN treatment (P<0.05), but there was no significant difference (P>0.05) between the control and the PMA plus TPEN group. PMA treatment significantly increased the activity of p34^cdc2^ kinase compared with control group (P<0.05). These data showed that PMA could rescue the low activity of p34^cdc2^ kinase from zinc depletion.

## Discussion

The role of zinc in meiotic division had been well demonstrated in MI and MII mouse oocytes [15], [18]. However, how zinc affects meiotic resumption in porcine oocytes is still unknown. In the present study, we found that the zinc content in the oocyte cytoplasm increases during meiotic division. Depletion of zinc abolished meiotic resumption, and this was rescued by treatment with a PKC activator. Depletion of zinc also reduced the phosphorylation of PKC substrates.

Our results showed that the level of zinc in porcine oocytes increased during maturation, which is in agreement with research on mice [23]. This previous report showed that the level of free intracellular zinc in oocytes during maturation is regulated by cumulus cells. Cumulus cells restrict increases in the level of zinc; however, this effect is abolished by treatment with epidermal growth factor (EGF) [23]. In the present study, the level of zinc increased in MII compared to GV stage oocytes, even though without supplement of zinc in the culture medium. These results showed that there must be presence of endogenous zinc in oocytes [18], and that has been released during oocyte maturation in response to EGF signaling pathway [23]. This zinc might be present in complex form, which is a concern of our further study. Moreover, the increase in the level of zinc during maturation indicates that zinc might be necessary for meiotic resumption.

To explore the function of zinc in meiotic resumption, zinc was depleted from porcine oocytes using TPEN. Depletion of zinc by TPEN in mouse oocytes reportedly accelerates GVBD [18]. However, different results were obtained with porcine oocytes. TPEN inhibited GVBD in a dose dependent manner, even when meiotic resumption was observed after 25 h of IVM. However, when oocytes were transferred to media lacking TPEN after 20 h of IVM, most oocytes subsequently developed to MII. These results revealed that zinc is involved in the molecular mechanism that regulates meiotic resumption. The differing effects of zinc depletion on mouse and porcine oocytes made us focus on molecules that regulate meiotic resumption, but that play different roles in mice and pigs. PKC isoforms comprise a family of protein kinases that play important roles in oocyte meiotic resumption. Lefevre et al. [27] showed that a PKC inhibitor can stimulate meiosis in mouse oocytes by microinjecting specific antibodies against different PKC isoforms, Avazeri et al. [9] further confirmed that PKC-βI and PKC-γ induce meiosis. Activation of PKC by treatment with PMA, a PKC agonist, increases meiotic resumption in pigs [10], whereas this process is completely blocked by treatment with the PKC inhibitor calphotin C [28]. Based on these results in mice and pigs, we hypothesized that a zinc dependent mechanism regulates meiotic resumption may via modulating PKC activity.

To prove this hypothesis, porcine oocytes were cultured in the presence of both TPEN and PMA. PMA treatment significantly reduced the percentage of oocytes at GV stage, even in the presence of TPEN. Given that PMA activates PKC, this result indicates that zinc affects the activity of PKC. This was supported by Western blot analysis of phosphorylated PKC substrates. Treatment with a low concentration of TPEN that did not affect oocyte maturation (1.0 or 2.0 µM) did not reduce phosphorylation of PKC substrates. By contrast, treatment with a concentration of TPEN that reduced oocyte maturation (2.7 or 3.0 µM) significantly reduced the level of phosphorylated PKC substrates. These data revealed that zinc depletion impaired meiotic division may by affecting PKC activity. PKC isoforms comprise a family of serine/threonine kinases that can be broadly categorized into three groups according to their cofactor requirements. Conventional PKCs (cPKCs, isoforms α, β, and γ) require Ca^2+^ and diacylglycerol (DAG) for maximal activity, novel PKCs (nPKCs, isoforms δ, ε, η, and θ) are Ca^2+^ independent but require DAG, and atypical PKCs (aPKCs, isoforms ζ, ι, and λ) require neither Ca^2+^ nor DAG for activity [29]. Activation of cPKCs and nPKCs requires phorbol esters (such as PMA), which are analogues of DAG and bind to the C1 domain (also known as the phorbol ester/DAG binding domain) of PKC [30]. The DAG/PMA binding domain binds to two zinc ions, which form the zinc finger [29]. Zinc depletion destroys the zinc finger in the C1 domain, thereby preventing binding of DAG and inhibiting the activation of cPKCs and nPKCs. aPKCs also have a zinc finger; however, the function of the zinc finger of aPKCs in oocytes is unknown.

Translation of *mos* mRNA is necessary to induce the MAP kinase cascade that indirectly activates MPF [31], a heterodimer of Cyclin B and Cdc2. MPF is responsible for many aspects of oocyte maturation, including GVBD [32]. In the current study, the levels of phosphorylated MAPK and MPF activity corresponded to the proportion of oocytes at GVBD stage. A low level of phosphorylated MAPK was rescued by PMA, which revealed that other mechanism regulate the content of MAPK. The mRNA levels of *C-mos*, *Cdc2*, and *CyclinB1* were significantly reduced in porcine oocytes cultured in the presence of TPEN, and this might underlie the reduced levels of MAPK and MPF. These changes in mRNA levels are probably owing to PKC related polyadenylation of mRNA. PKC indirectly regulates polyadenylation. In immature oocytes, activated PKC inactivates glycogen synthase kinase (GSK)-3. GSK-3 phosphorylates Aurora A on residues S290/S291, which, in turn, stimulates inhibitory autophosphorylation of Aurora A on residue S349. Active Aurora A phosphorylates CPEB on residue S174, which induces polyadenylation and translation of mRNA [33]. The influence of the length of the poly(A) tail on mRNA stability is well known. Structural analysis revealed that CPEBs are zinc binding proteins [34]. In most cases, the zinc fingers of CPEBs mediate their binding to DNA [35], [36] or RNA [34]. It is likely that zinc depletion not only regulates polyadenylation via the PKC-related pathway, but also directly reduces binding between CPEBs and mRNA [34] by destroying the zinc fingers. The reduction in MPF activity following zinc depletion causes the release of APC from EMI2 and further increases MPF degeneration [18], [37]. Our results indicate that perturbation of MPF synthesis also underlies why the level of MPF is reduced in the absence of zinc.

The reduction in MPF activity following TPEN treatment was completely rescued by PMA, whereas MAPK phosphorylation was only partially rescued. Moreover, MPF activity was higher in the TPEN plus PMA and PMA groups than in the TPEN group. This revealed that the level of MPF degradation in oocytes was low. Prevention of MPF degeneration by EMI2 requires zinc; however, how to supply sufficient zinc in the TPEN content medium is uncertain. Surprisingly, the level of cytoplasmic zinc sharply increased following PMA treatment to a level similar to that in MII, showing that zinc efflux is related to PKC activation. The structure of PKC has been compared before and after activation by PMA or DAG. Purified recombinant PKC protein fragments shed stoichiometric amounts of zinc from their zinc finger domains following activation [38]. However, aPKCs are not reportedly activated by PMA or DAG *in vitro* or *in vivo* [39]. These results suggest that cPKCs and nPKCs are the main sources of zinc in oocytes. This indicates that activation of PKCs is important to ensure the normal physiology of oocytes, not only directly by regulating several signaling pathways, but also indirectly by ensuring that there is a sufficient supply of zinc.

In the present study, after PKC activation, significantly zinc content increasing was observed, however, depletion of zinc reduced the activity of PKC. PKC and zinc might regulate each other. Korichneva et al. (2012) reported that zinc released from PKC is a common event after PKC activation [38], showed that PKCs might regulate zinc content in cytoplasm, and the releasing ensured sufficiency zinc in cytoplasm. From another point of view, low concentration of zinc reduced the activity of PKC in the present study. Depletion of zinc by TPEN also prevent the activation and translocation of PKC [40]. It revealed that zinc content is very important for maintaining PKC activity.

In conclusion, zinc regulates meiotic resumption in mammalian oocytes via modulating activation of PKC, which, in turn, affects activation of MAPK and MPF. Activation of PKC increases the level of zinc in oocytes.
