# Expression of Concern: Zinc Regulates Meiotic Resumption in Porcine Oocytes via a Protein Kinase C-Related Pathway

**DOI:** 10.1371/journal.pone.0221306

**Published:** 2019-08-13

**Authors:** The PLOS ONE Editors

After publication of this article [1], concerns were raised about Fig 1A having an overlapping region between the top of the 28 h panel and the bottom of the 44 h panel, though the two panels appear to show different fluorescence intensity.

The reported methodology for Fig 1A indicates oocytes were cultured for the different time periods in IVM medium, and then at the stated time points they were stained and mounted for immediate imaging. The reported results of the experiment show an increase in fluorescence intensity over time. The corresponding author has stated there was an error in selecting the images when assembling Fig 1A and acknowledges that the 28 h and 44 h panels show images taken in different areas of the same dish. Regarding the difference in fluorescence intensity, the corresponding author has explained that due to the very low fluorescence intensity at 0 h, several different exposure parameters were explored to obtain the best display effect; a single parameter was then selected to represent all groups. As a result, many images were collected under different parameters, and the wrong image was selected for inclusion in Fig 1A.

The authors have repeated the experiment and provide a revised Fig 1 based on the new data, along with the underlying data and image files, as Supporting Information (S1 File)

The authors also provide the available underlying data for the other figures as Supporting Information. The original data is now missing for Fig 5C, Fig 6A and the chart and the Actin panel of the western blot in Fig 6B, and Fig 7.

The *PLOS ONE* Editors issue this Expression of Concern to alert readers to concerns about the data handling and figure preparation, and the unavailability of parts of the underlying data.

## Supporting information

S1 File Revised Fig 1 and available underlying data. (ZIP)
