# Supplementary figures and images for: Expression of Concern: Zinc Regulates Meiotic Resumption in Porcine Oocytes via a Protein Kinase C-Related Pathway
Source: PLoS One. 2019 Aug 13;14(8):e0221306. doi: 10.1371/journal.pone.0221306 (PMC6692020; doi:10.1371/journal.pone.0221306)

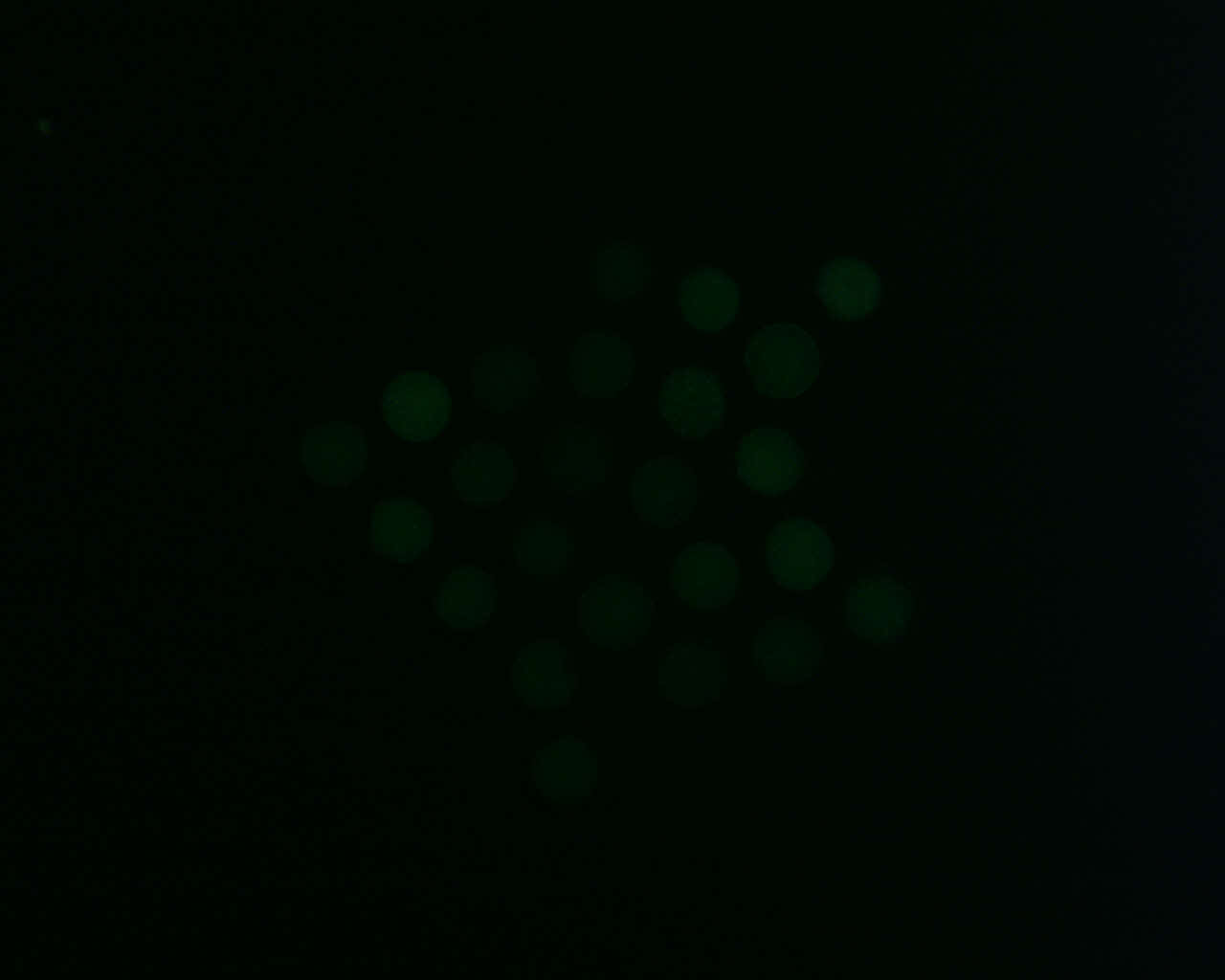

Supplement: S1 File — (ZIP) [file pone.0221306.s001.zip › Raw data (2)/Figure 1 New Repeat/0h.jpg]

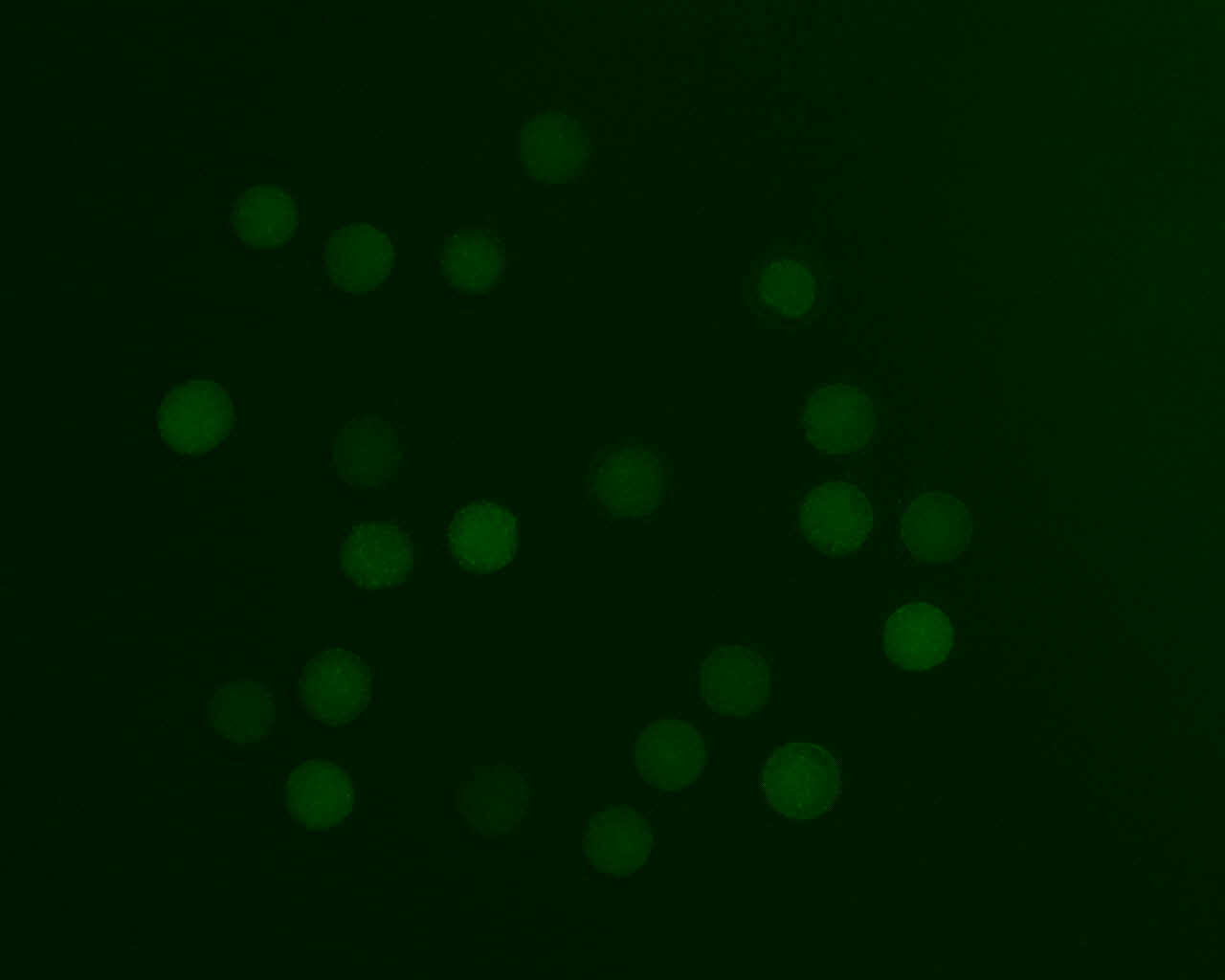

Supplement: S1 File — (ZIP) [file pone.0221306.s001.zip › Raw data (2)/Figure 1 New Repeat/20h.jpg]

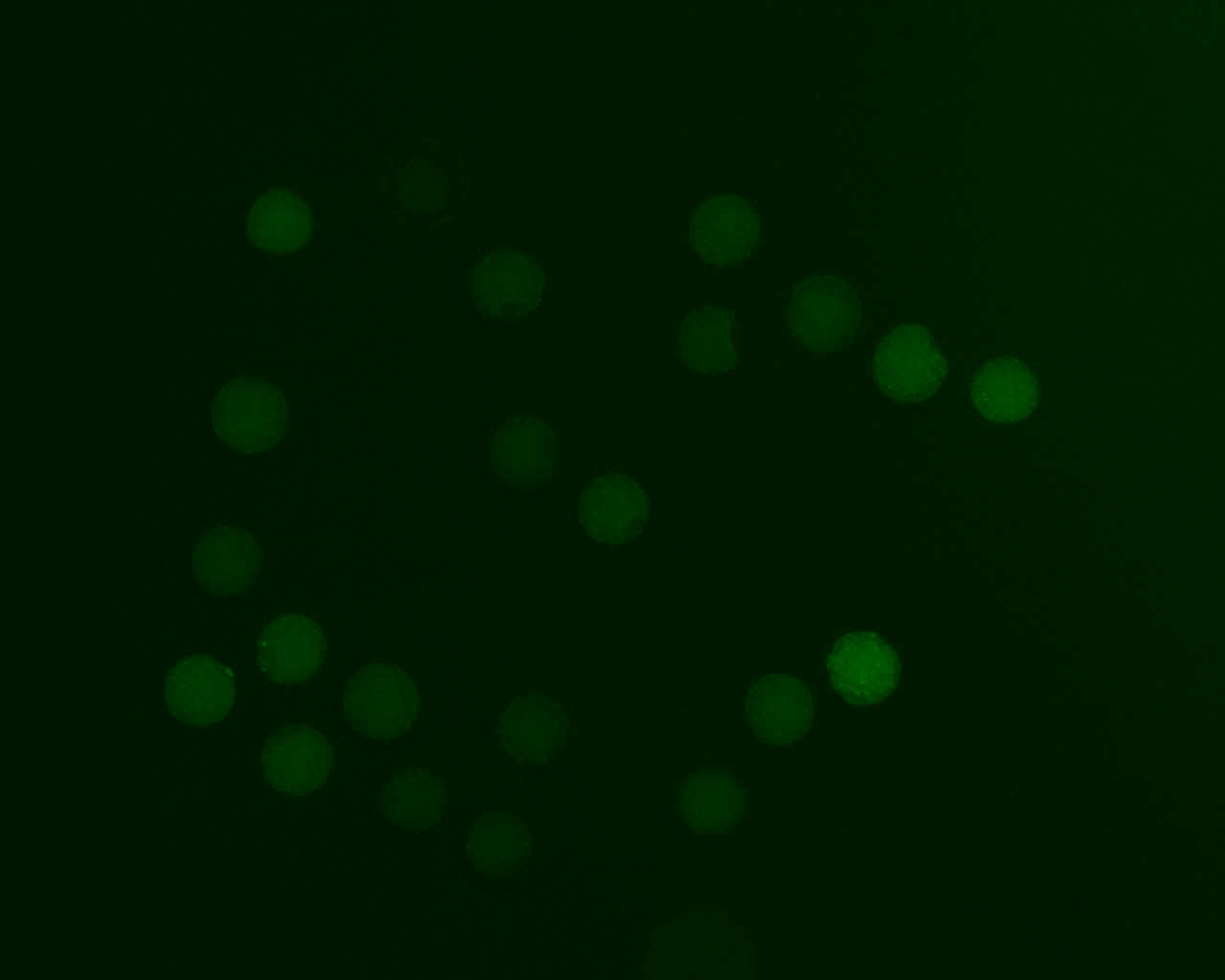

Supplement: S1 File — (ZIP) [file pone.0221306.s001.zip › Raw data (2)/Figure 1 New Repeat/28h.jpg]

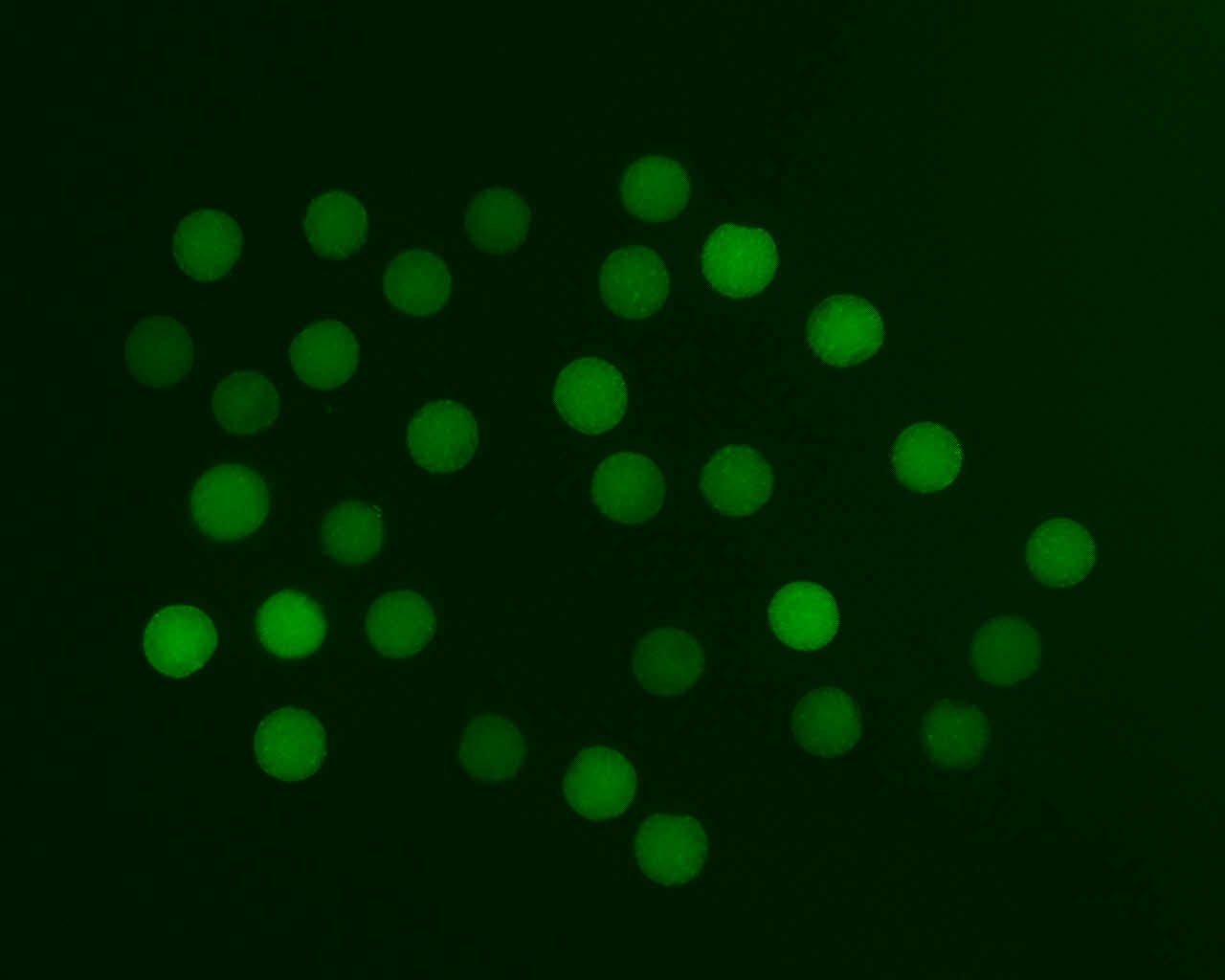

Supplement: S1 File — (ZIP) [file pone.0221306.s001.zip › Raw data (2)/Figure 1 New Repeat/44h.jpg]

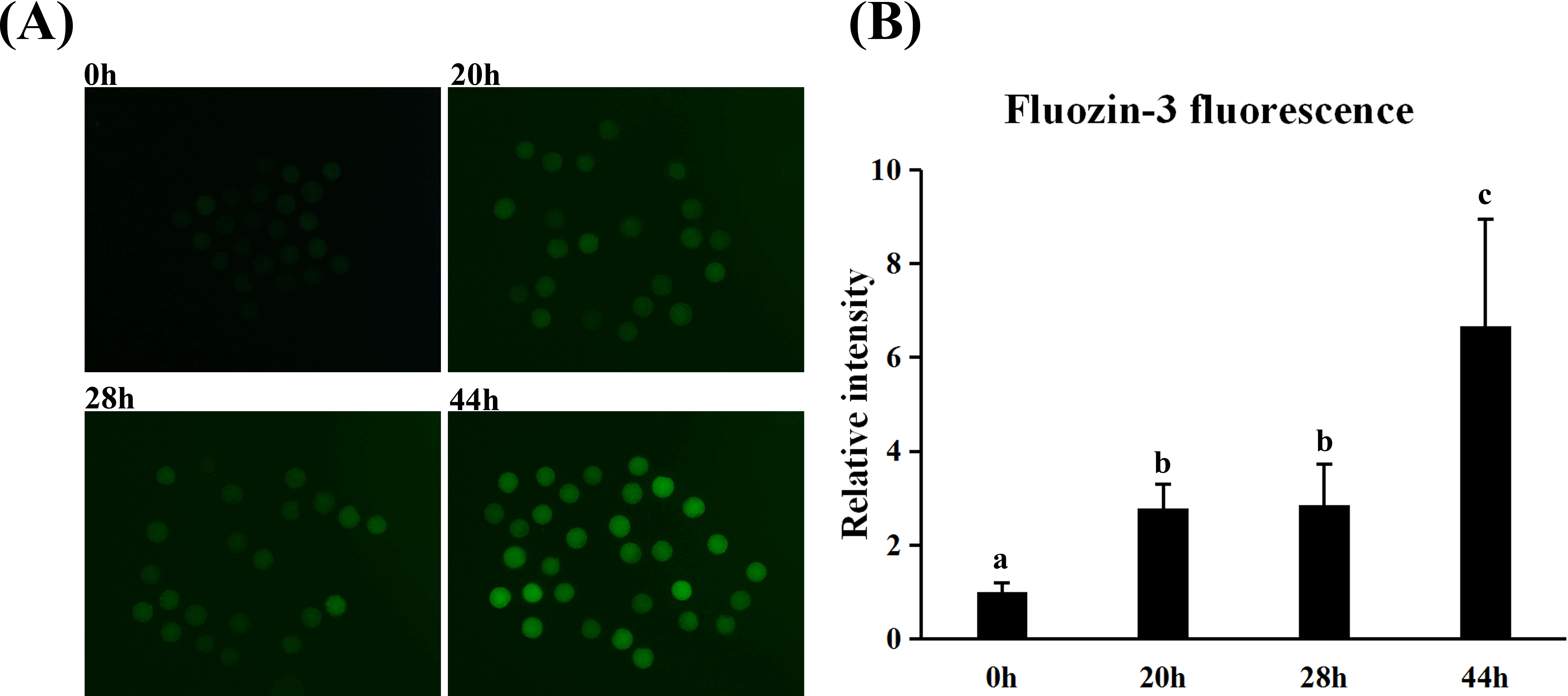

Supplement: S1 File — (ZIP) [file pone.0221306.s001.zip › Raw data (2)/Figure 1 New Repeat/Fig1.tif]

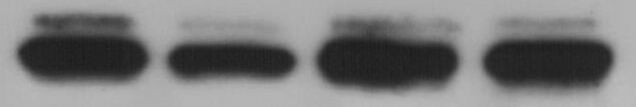

Supplement: S1 File — (ZIP) [file pone.0221306.s001.zip › Raw data (2)/Figure 6/Figure 6A-pERK12.jpg]

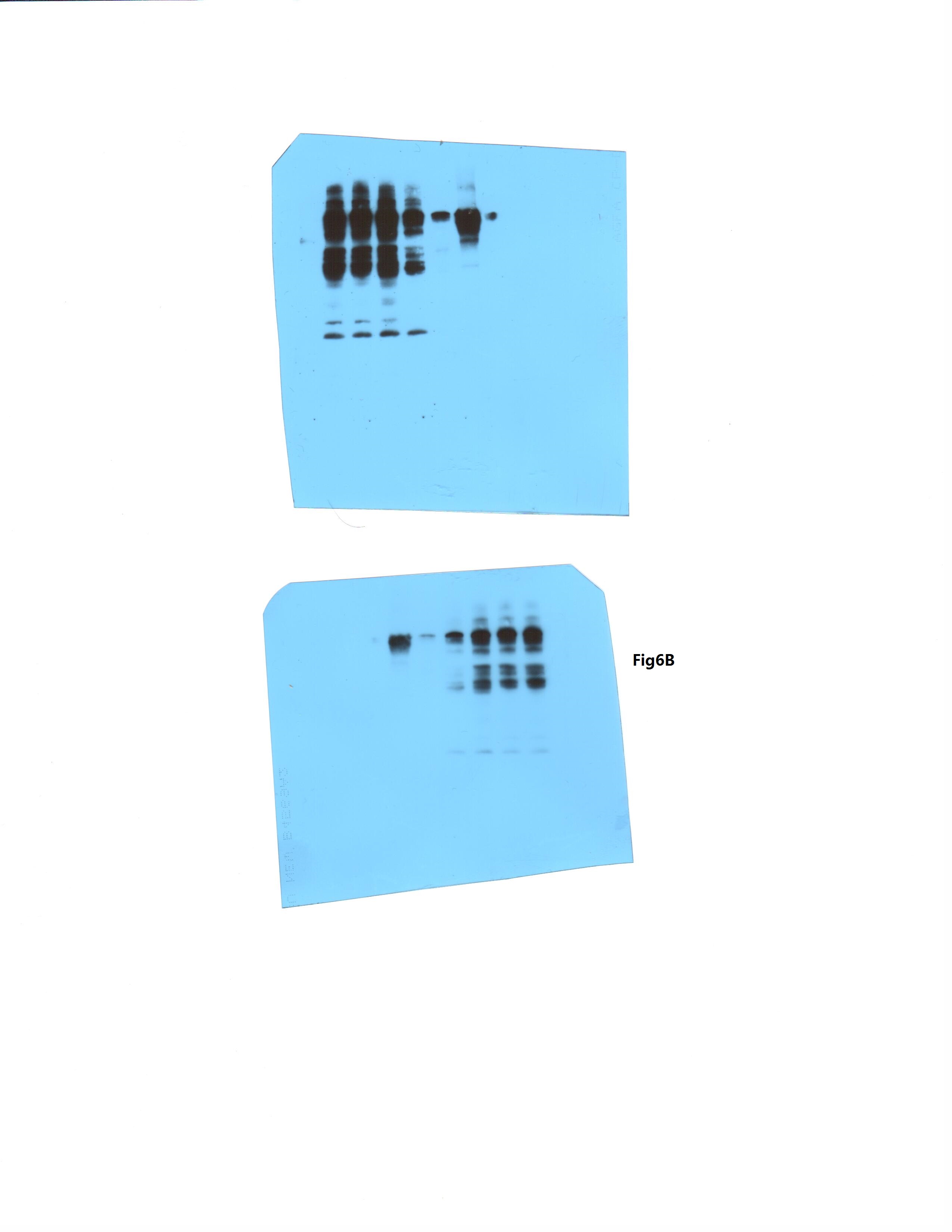

Supplement: S1 File — (ZIP) [file pone.0221306.s001.zip › Raw data (2)/Figure 6/Figurea 6B.jpg]
